# Role of whole bone marrow, whole bone marrow cultured cells, and mesenchymal stem cells in chronic wound healing

**DOI:** 10.1186/s13287-015-0001-9

**Published:** 2015-03-13

**Authors:** Luis Rodriguez-Menocal, Shahjahan Shareef, Marcela Salgado, Arsalan Shabbir, Evangelos Van Badiavas

**Affiliations:** Department of Dermatology and Cutaneous Surgery, Leonard M. Miller School of Medicine, University of Miami, Interdisciplinary Stem Cell Institute, 1501 NW 10th Street, Suite 924, Miami, FL 33136 USA

## Abstract

**Introduction:**

Recent evidence has shown that bone marrow cells play critical roles during the inflammatory, proliferative and remodeling phases of cutaneous wound healing. Among the bone marrow cells delivered to wounds are stem cells, which can differentiate into multiple tissue-forming cell lineages to effect, healing. Gaining insight into which lineages are most important in accelerating wound healing would be quite valuable in designing therapeutic approaches for difficult to heal wounds.

**Methods:**

In this report we compared the effect of different bone marrow preparations on established in vitro wound healing assays. The preparations examined were whole bone marrow (WBM), whole bone marrow (long term initiating/hematopoietic based) cultured cells (BMC), and bone marrow derived mesenchymal stem cells (BM-MSC). We also applied these bone marrow preparations in two murine models of radiation induced delayed wound healing to determine which had a greater effect on healing.

**Results:**

Angiogenesis assays demonstrated that tube formation was stimulated by both WBM and BMC, with WBM having the greatest effect. Scratch wound assays showed higher fibroblast migration at 24, 48, and 72 hours in presence of WBM as compared to BM-MSC. WBM also appeared to stimulate a greater healing response than BMC and BM-MSC in a radiation induced delayed wound healing animal model.

**Conclusions:**

These studies promise to help elucidate the role of stem cells during repair of chronic wounds and reveal which cells present in bone marrow might contribute most to the wound healing process.

## Introduction

Conditions such as diabetes, chronic renal failure, arterial or venous insufficiency, and radiation-induced tissue damage are among the multifactorial processes that contribute significantly to dysfunctional wound healing [1-3]. These complex wounds are characterized by inhibition of the inflammatory response, dysfunctional macrophages leading to an inability to combat infection, impaired angiogenesis and vasculogenesis, accumulation of fibrous tissue, and aberrant extracellular matrix accumulation [4]. Numerous therapies have been attempted to treat chronic wounds. Approaches promoting healing such as debridement, frequent dressing changes, antibiotic therapy, and increasing tissue growth factor levels have proven to be of limited efficacy [3,4].

Recent studies have shown that the regenerative potential of stem cells may be applicable to the treatment of healing chronic wounds [5]. Using somatic stem cells rather than embryonic stem cells paves the way for a treatment that is limited in ethical concerns. Bone marrow has been used as a source of cellular therapy because it contains inflammatory cell progenitors long identified as being important in wound healing as well as mesenchymal stem cells, and other multipotent stem cells [6]. Mesenchymal stem cells have the potential to rebuild the dermis by differentiating into many cell types such as fibroblasts, cartilage and muscle [3]. These cells can also release many growth factors and cytokines that are vital to wound repair. Other multipotent cells, such as hematopoietic stem cells and vascular progenitors, are also present in bone marrow and likely contribute significantly to wound repair [7].

The multipotent capability of bone marrow cells gave an impelling reason to study the role of bone marrow in chronic wound healing and several clinical studies have reported on its benefit [8]. Studies comparing these preparations are, however, needed in order to begin examining which cell types and preparations may be most beneficial in designing improved treatment protocols. We have utilized mouse models to investigate the effectiveness of whole bone marrow (WBM), whole bone marrow (long term initiating/hematopoietic based) cultured cells (BMC), and bone marrow derived mesenchymal stem cells (BM-MSC) in both *in vitro* and *in vivo* murine wound healing models. The *in vitro* models we studied included angiogenesis and scratch migration assays. For *in vivo* models we utilized two models of radiation induced delayed wound healing. In the more standard model, split dose not ablative radiation was administered to animals prior to creating wounds and administering cells. In another ‘reverse’ model, wounding was performed between the split doses of non-ablative radiation and cells applied after all radiation doses were given. Wound healing was delayed to a greater extent in the reverse model. The availability of transgenic C57/BL6 mice expressing GFP in all tissues provides the opportunity to deliver and track donor cells in non-GFP recipient mice. The percentage of wound closure, engraftment and stimulation of wound healing were among the endpoints evaluated.

## Methods

### Mice and isolation of whole bone marrow (WBM)

Recipient four-week-old female C57BL/6 mice and donor male GFP^+^ transgenic C57BL/6(SJL)-Tg(UBC-GFP,-TVA)1Clc/J mice were obtained from Jackson Laboratories, Bar Harbor, ME, USA. The Institutional Animal Care and Use Committee approved the experimental protocol for these studies. Isolation of whole bone marrow cells from compact bone of C57BL/6-Tg (UBC-GFP) mice was done using the protocol provided by Stem Cell Technologies Inc., Vancouver, Canada. Cat No 28453. After the whole bone marrow was isolated it was cryopreserved and placed in liquid nitrogen freeze storage tanks.

### Whole bone marrow cultured cells

The WBM cultures utilized here are long term initiating cultures, which are capable of retaining functional primitive hematopoietic stem and progenitor cells for many weeks [9]. To create these cultures, freshly isolated WBM from GFP^+^ transgenic C57BL/6-Tg (UBC-GFP) male donor mice was cultured as previously described [2]. Cultures were incubated at 33°C and 5% CO_2_ in 225 cm^2^ Corning flasks and were fed weekly without removing any of the previous media. These cultures have been demonstrated to have clinical benefit in treating recalcitrant chronic wounds in patients [2]. In order to be consistent with previous clinical studies, these cells were harvested fresh for each experiment after four weeks of culture.

### Bone marrow derived mesenchymal stem cells

Mesenchymal stem cells were generated according to the protocol described by Stem Cell Technologies Inc. Cat No 28453. These methods do not support the long-term growth of hematopoietic stem and progenitor cells. WBM derived from C57BL/6-Tg (UBC-GFP) mice was placed into selective media (Complete MesenCult© Medium - Stem Cell Technologies Inc. Cat. No 05501, 05502) allowing the growth of mesenchymal stem cells. The cells were incubated in a 225 cm^2^ Corning flask at 37°C and 5% carbon dioxide for 10 days. After that half of the medium was changed and replaced with an equal volume of Complete MesenCult© Medium. When the cells reached approximately 80% confluence they were expanded (expansion 1:3) to reach passage four (P4). An Olympus fluorescent microscope and Metamorph Software were used to determine if the BMC and BM-MSC were healthy and expressing GFP. After five weeks of culture, cells were harvested, counted, cryopreserved and placed in nitrogen freezing storage tanks. In clinical protocols utilizing these cells, they are typically administered from cryopreserved materials [10].

### Human umbilical vein endothelial cells

A human umbilical vein endothelial cell (HUVEC) line and culture medium were obtained from Invitrogen Catalogue/Life Technologies-NY, USA. No A1460901.

### *In vitro* angiogenesis assay

HUVEC suspensions were seeded in 24-well plates containing Geltrex^TM^ matrix (Invitrogen) and incubated for 30 minutes at 37°C. Boyden chamber transwell inserts of 8 μm containing 10^4^ cells corresponding to WBM, BMC, BM-MSC and controls were then placed in individual HUVEC seeded wells. In this experiment we used Medium 200 containing 2% (v/v) fetal bovine serum (FBS) and basic fibroblast growth factor (bFGF, 3 ng/μl) as the positive control and Medium 200 (Invitrogen) as the negative control. The 24-well plates containing inserts were incubated at 37°C in a humidified atmosphere of CO_2_ in air for eight hours. The Boyden chamber transwells containing bone marrow cells were then removed and the wells containing HUVEC cells were stained for 30 minutes with calcein-AM dissolved in dimethyl sulfoxide (DMSO). The wells were then photographed using an inverted IX81 Olympus microscope (Olympus America, Center Valley, PA, USA) and ORCA-AG Hamamatsu digital camera (Hamamatsu Photonics K.K., Hamamatsu City, Shizuoka Pref., Japan) and tube formation was assessed using Image J software.

### Cell migration assay

The cell migration assay was performed using a modified Boyden chamber (8 μm pore size) [11]. First, the mouse fibroblasts were seeded onto 24-well dishes at a density of 10^4^ cells per well. The dishes were incubated to establish confluent monolayers and then treated with 10 mg/ml mitomycin for two hours before scratch–wounding. Cell monolayers were mechanically disrupted with a 1 mL pipette tip. After disruption, monolayers were gently washed twice with PBS to remove cell debris. Serum-free media (300 μl) was added to these wells (lower chamber) containing mouse fibroblast cells. Subsequently, 10^4^ cells corresponding to WBM, BMC or BM-MSC were seeded at the top of the Boyden chamber insert in 200 μl of serum free medium and placed into wells containing scratch-wounded fibroblasts. Wells containing 200 μl of medium without cells were used as the negative control. The width of the denuded surface between the edges of defect was measured immediately after disruption and after 24, 48 and 72 hours. Defect closing was documented photographically (five pictures per well) using the inverted IX91 Olympus microscope described above. The unclosed distance (μM) between two edges at three different points along the length of the defect was quantified as an average gap.

### Wound healing model

C57BL/6 mice were used for this experiment. All mice (n = 12) were radiated for five minutes at 80 rad/minute in order to reduce their rate of wound closure and allow for better engraftment of delivered cells. After a four-hour resting stage the mice were again radiated for five minutes at 80 rad/minute to maximize these effects. Mice were anesthetized by i.p. injection of 87 mg/kg ketamine and 13 mg/kg xylazine, prior to all procedures. To create wounds, the back of the mice closest to the tail was shaved and an 8 mm full thickness wound was made using a punch biopsy instrument (Sklar Surgical Instruments Corp.-PA, USA). All cells were administered at a dose of 2 × 10^6^ cells via tail vein injection. Control mice were irradiated and wounded but not injected with any cells. Classical and Reverse models differed in the timing of irradiation, wounding and cell administration. In the Classical model, mice were first irradiated two times, rested for 24 hours, administered cells by tail vein injection, rested (to allow for stem cell engraftment) for 30 days and then wounded. For the Reverse model, mice were irradiated once, wounded, irradiated the second time, rested 24 hours and then administered cells. Each group contained four wounded and irradiated mice.

### Morphometric analysis of wounded mice

At time zero, digital photographs were taken as soon as the punch biopsy wound was made on the back of the mice in order to establish a baseline. Photographs were taken again on days 2, 4, and 7 and analyzed using the cellSens Standard software (Olympus Corporation) to determine the percentage of wound closure by conversion of pixels to micrometers. The percentage of wound closure was averaged for each treatment group.

### FACS analysis

In order to establish that the animals in the classical model retained engrafted cells at the time of wounding, assessment of engraftment of bone marrow cells was evaluated in peripheral blood collected from the tail veins of mice 28 days after receiving bone marrow cells. Non-transplanted C57BL/6 and transgenic C57BL/6-Tg (UBC-GFP) mice served as positive and negative controls. Heparinized mouse blood (0.3 mL) was centrifuged at 1,400 g for five minutes at 4°C, and each blood sample was incubated with 3 mL of NH_4_Cl, pH 7.2, for ten minutes at room temperature to lyse the red blood cells. The remaining leukocytes were washed with phosphate-buffered saline (PBS), pH 7.4, and flow cytometry was carried out in the fluorescein isothiocyanate (FITC) channel. Fluorescence-activated cell sorting (FACS) analysis of each mouse blood sample was performed to evaluate the level of GFP chimerism. Five mice for each group were used for FACS analysis. The results show that more than 60% chimerism was obtained one month after cells were transferred via tail vein injection.

### Statistical analysis

To statistically analyze differences between multiple groups at one time point, one-way analysis of variance (ANOVA) with its subsequent Bonferroni’s multiple comparison tests were used. Graphs and statistics were generated using Prism version 5 for Mac OS X. A value of *P* of less than 0.05 was considered statistically significant. The quantitative results are expressed as mean +/− SEM (standard error of the mean).

## Results

### Contribution of bone marrow cells in a matrigel tube formation assay

**Figure 1:**
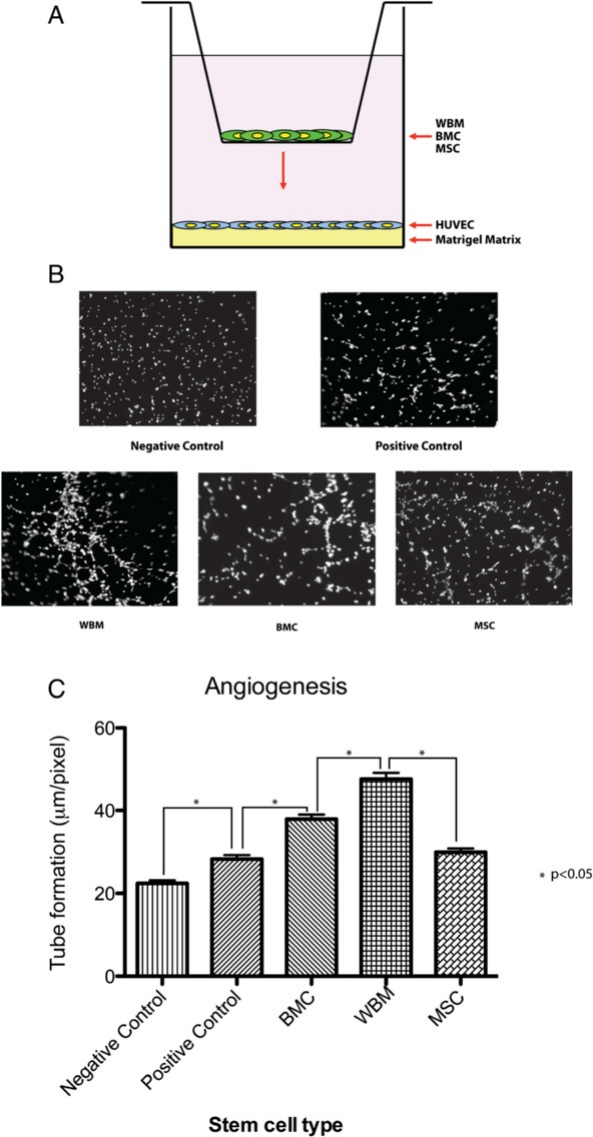
**Matrigel tube formation assay. (A)** Schematic diagram of the tube formation assay chamber. HUVECs were seeded in 24-well plates containing Geltrex^TM^ matrix in the lower chamber. The upper chamber was prepared by plating a monolayer of BMC, WBM or MSC onto a very thick layer followed by incubation at 37°C for eight hours. Following incubation, the Boyden chamber containing the cells was removed and the plate with HUVEC cells was stained for 30 minutes with calcein-AM dissolved in DMSO. The number of tube-like structures that formed in the gel was measured by total length per field (X 200). **(B)** Phase contrast micrographs show HUVECs grown in matrix gel and tubular structure induced by different kinds of stem cells: (I) negative control (Medium 200); (II) positive control (Medium 200 containing 2%(v/v) FBS and bFGF (3 ng/μl); (III) BMC: whole bone marrow cultured cells; (IV) whole bone marrow and (V) mesenchymal stem cells. **(C)** Quantitative analysis of angiogenesis *in vitro* by measuring the occupied area due to the tubular structures formed by HUVECs. Photographs were taken eight hours after the HUVECs were seeded and inserts containing the stem cells were placed in the 24 well plates. In this experiment we used Medium 200 containing 2% (v/v) FBS and bFGF (3 ng/μl) as the positive control and Medium 200 from Invitrogen as the negative control. The induction of matrigel tube formation is higher in the presence of WBM compared with BMC and MSC. WBM versus BMC *P* <0.05, WBM versus MSC *P* <0.05. Data are expressed as mean +/− SEM of five measurements. bFGF, basic fibroblast growth factor; BMC, whole bone marrow cultured cells; DMSO, dimethyl sulfoxide; FBS, fetal bovine serum; HUVECs, human umbilical vein endothelial cells; MSC, mesenchymal stem cells; SEM, standard error of the mean; WBM, whole bone marrow.

Wound tissue regeneration is highly dependent on proliferation of local cells and blood supply. The angiogenic support provided by stem cells in this process is fundamental in the reestablishment of blood supply for recovery of damaged tissue. We utilized an *in vitro* tube formation assay to analyze the contribution of these stem cell preparations in tissue repair (Figure 1A); for this purpose, HUVEC cells were seeded in matrigel and bone marrow cells (as described above) were added. After eight hours, we analyzed the tube formation stimulated by WBM, BMC, BM-MSC and negative control (no bone marrow cells added). We found that tube formation was stimulated by both WBM and BMC with WBM having the greater effect. The effect noted by adding BM-MSC appeared to be much smaller and comparable to the positive control (Figure 1B).

Figure 1C shows a qualitative representation of tube formation when the branch number is higher in the presence of WBM compared to BMC and MSC.

### Contribution of whole bone marrow compared with MSC in a scratch assay

**Figure 2:**
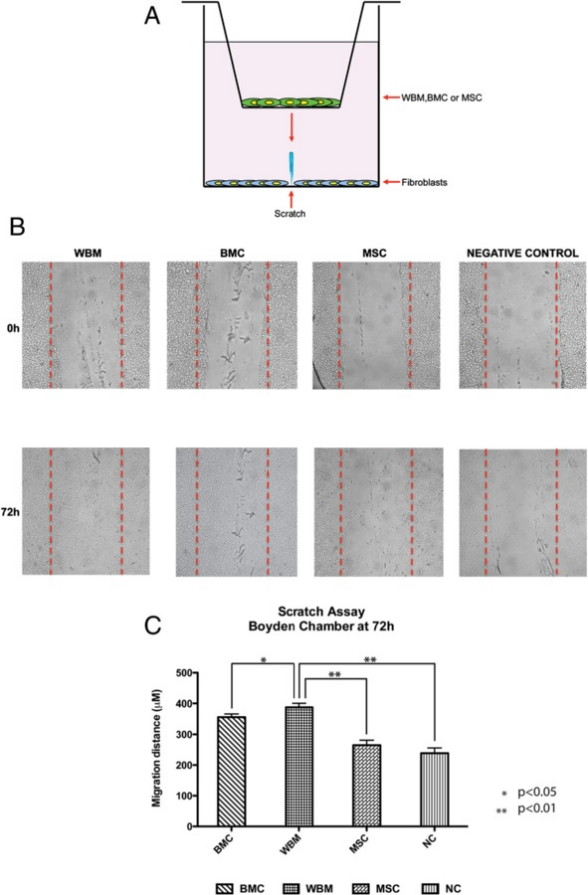
**Effects of WBM, BMC and MSC on fibroblast migration in a ‘wound’ scratch assay. (A)** Schematic diagram of the transwell migration assay: Fibroblasts cells were plated in the lower chamber. Cell monolayers were mechanically disrupted with a 1 mL pipette tip. In the upper chamber, the cells (WBM and MSC) were allowed to migrate for 24, 48 and 72 hours toward the lower chamber. Photographs were taken at 0, 24, 48 and 72 hours to examine the defect in closing. **(B)** Photographs of wounded mouse fibroblast monolayers at 0, 24, 48 and 72 hours after wounding. **(C)** Scratch assays quantification in a wound-healing model. Effects of bone marrow derived cells on mouse fibroblast migration in a wound scratch assay. In order to determine the percentage scratch closure the cell free space was measured using cellSens software installed in an Olympus microscope and pixels were converted to micrometers. The migration value was calculated as wound closure distance from time zero to 24, 48 and 72 hours respectively (∆d = d_t0_-d_72h_). The *in vitro* wound-healing assay showed that WBM strongly improved the fibroblast wound closure compared with BMC, MSC and the control group. In this experiment the data represent mean +/− SEM of five measurements. BMC, whole bone marrow cultured cells; MSC, mesenchymal stem cells; SEM, standard error of the mean; WBM, whole bone marrow.

Cell migration is a crucial event in cutaneous wound repair and understanding how stem cells influence cell migration will aid in developing protocols for improved wound healing. To examine whether MSC, WBM or BMC promote cell migration during wound closure, we performed an *in vitro* scratch wound assay as described above (Figure 2A). The distance between wound edges was measured at 0, 24, 48 and 72 hours to determine migration values (migration values = D_0_-D_T_). In the scratch wound assay represented in Figure 2B and 2C we observed higher fibroblast migration in the presence of WBM or BMC as compared to MSC (*P* <0.01). Differences between WBM compared to BMC were also noted to be significant (*P* <0.05) with WBM showing a greater effect (Figure 2C).

### Classical versus reverse model for wound healing in mice

**Figure 3:**
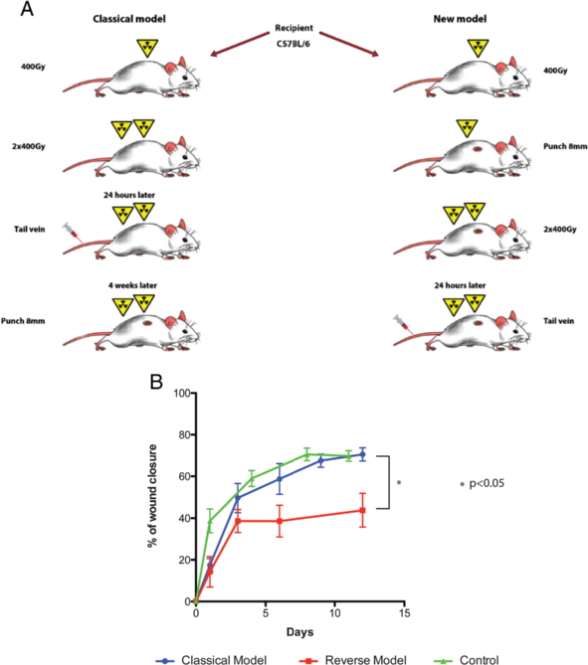
**Classical and reverse model of wounding procedure in mice. (A)** Diagram with classical and reverse wound model procedure**.** In the classical model the 8 mm punch is made 30 days after irradiation and in the reverse model the 8 mm punch is created between the two cycles of radiation. **(B)** Wound healing is expressed as percentage closure relative to original size (1-(wound area on day x)/(wound area on day 0)) x100. The reverse model showed lower percentage closure at all time points compared to the classical model. *P* <0.05 indicates a statistically significant difference in wound area between the reverse and classical models. All values are expressed as the mean and SEM. Each group comprised 12 animals (n = 12). SEM, standard error of the mean.

In both the classical and reverse models we used fractioned total body irradiation with a four hour interval between doses. A significant difference between the classical and reverse wound healing models is that in the classical model an 8 mm punch biopsy excision is made 30 days after the last radiation dose whereas in the reverse model the 8 mm punch is created between the two cycles of radiation. We found that wound closure for both models was delayed in comparison to control (untreated) mice and that wounds in the reverse model were significantly delayed in closure compared to the classical model. This likely is due to a more direct effect of radiation on reducing available resident stem cells to orchestrate repair following injury. The reverse model, therefore, promised to give us a greater ability to examine the effects of delivered stem cells on wound closure (Figure 3B).

### Stem cells contribution to wound healing in a mouse model

**Figure 4:**
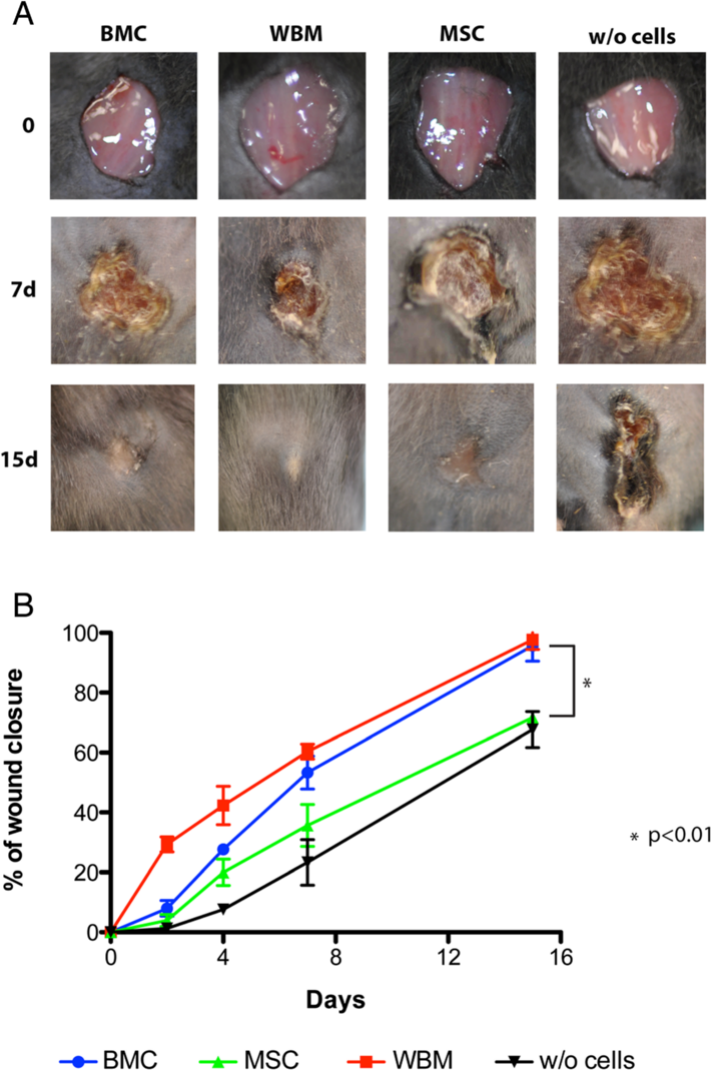
**Effects of WBM, BMC and MSC on the** ***in vivo*** **wound closure in the mouse model and analysis of wounds. (A)** Representative photographs of the mouse excisional reverse model after transplantations of BMC, WBM, MSC and control vehicle at 0, 7 and 15 days. **(B)** Epithelialization for each group after transplantations of BMC, WBM, MSC and control vehicle is expressed as percentage closure relative to the original size (1-(wound area on day x)/(wound area on day 0)) x100. WBM treated wounds featured greater percentage closure at all time points compared to BMC and MSC. Data represent mean +/− SEM of five measurements. Each group comprised 12 animals (n = 12). *P* <0.01 indicates a statistically significant difference in wound closure between animals treated with WBM or BMC compared to animals treated with MSC and control group. BMC, whole bone marrow cultured cells; MSC, mesenchymal stem cells; SEM, standard error of the mean; WBM, whole bone marrow.

The reverse model gave us a unique opportunity to test the ability of our three cell preparations to overcome the significant delay in wound healing observed,

Treatment with WBM, BMC and BM-MSC showed improvement in wound closure over controls (Figure 4A). At Day 7, all cell preparations appeared to improve wound closure significantly over control with BMC and WBM showing the greatest effect (Figure 4B). At Day 15, BMC and WBM treated wounds were completely epithelialized while wounds treated with MSC or given no cells were still not fully epithelialized. The slight improvement in WBM over BMC might be due to the increased amounts of cytokines in a whole tissue preparation. However, this degree of similarity in wound closure indicates that stem and progenitor cells important for skin repair are not lost in the process of cell culture [5]. Epithelialization of wounds was at first (Day 2) fastest in animals treated with WBM and later (Day 7) in animals treated with BMC (Figure 4B). This indicates that combinations of varied stem and progenitor cells are important in initiating wound repair and that single stem cell types (here BM-MSC) may be less effective. This initiation of wound repair could be critical in reversing the phenotype of chronic wounds where stem and progenitor cells appear to be reduced or functionally depleted [12,13]. Overall, the results from these experiments support that bone marrow has stem and progenitor cells that can stimulate wounds and contribute to healing [3,5].

## Discussion

The multifunctional properties of bone marrow-derived cells, including their ability to differentiate into various cell types and capacity to secrete factors important in accelerating healing of cutaneous wounds, have made the search for a particular stem cell to orchestrate repair an important goal. Mesenchymal stem cells derived from bone marrow have become a promising agent for tissue repair and regeneration and are the focus of numerous clinical studies. It has been reported that BM-MSC could act as a chemo-attractant for macrophages and endothelial cells and recruit dermal fibroblasts to the wound site by paracrine signaling [12].

Wounding, however, stimulates the recruitment of several types of bone marrow derived cells to the skin including inflammatory cells and vascular progenitor cells. Some recent evidence supports that bone marrow might also serve as a resource to provide skin progenitor cells [5]. Given the uncertainty of which bone marrow cells might be required at each phase of tissue repair and regeneration, a mixed cell approach might then be more useful. To help answer these questions, we began by examining three bone marrow cell preparations in two *in vitro* assays examining angiogenesis and migration.

During the early stages of skin wound healing, angiogenesis is fundamental for the effective development of dermal tissue and is a hallmark of an established healing response. With new blood vessel growth, steps involving dissolution of basement membrane underlying endothelial cells, endothelial cell migration, adhesion, proliferation and tube differentiation are required [14-16]. Moreover, the vasculature comprises up to 60% of repair tissue in a wound, and the newly established blood supply is required to meet the metabolic demands of wound repair as well as to deliver needed repair cells from distant sites. All these elements support the importance of angiogenesis and vascularization in wound repair. In our studies we examined three clinically utilized cell preparations to evaluate their role in wound repair. We found that WBM was able to achieve higher new vessel tube formation compared with BMC and MSC. This implies a potential significant advantage for WBM to stimulate endothelial cells and angiogenesis in tissue repair.

Cell migration is another crucial event in cutaneous wound healing. Secreted factors are certain to be critical in stimulating cell migration and, if optimized, could be an essential component in developing improved therapeutics for wound healing. The *in vitro* scratch wound assays using WBM, BMC and BM-MSC demonstrated that paracrine signaling from bone marrow cells influences dermal fibroblast migration. Here again, WBM appeared superior to cultured cells. It could be that WBM, having a greater mixed population of cell types than BMC or BM-MSC, was capable of producing a more wide-ranging cytokine profile that more closely resembled that seen in healing wounds. Cultured bone marrow cells, however, have shown benefit in chronic wounds [2]. Dermal cells derived from these wounds typically demonstrate evidence of senescence and lack of response to growth factors [17,18]. We have previously shown that BM-MSC stimulates chronic wound fibroblast to migrate [12]. The murine base scratch assay performed in this study might not be representative enough of human chronic wound fibroblasts to fully evaluate the effect of cultured bone marrow cells on difficult to heal wounds.

To examine the effect of WBM, BMC and BM-MSC *in vivo*, we investigated two murine models of delayed wound healing. Epithelialization, granulation tissue formation, scar formation, contraction and angiogenesis are seen in both humans and mice. Wound healing in mice, however, occurs quite rapidly due in part to their greater ability to heal by contraction [19,20]. Human chronic wounds, unlike wounds in mice, are significantly reduced in resident stem cells [21]. Patients with chronic wounds also have been shown to have abnormalities in their bone marrow cells [2,10,22]. In this study, we wished to create a murine model that would be depleted of resident stem cells and demonstrate prolonged wound closure. Gamma irradiation of mice has been used as an effective tool for depleting stem cells [23,24]. Fractionated total body irradiation has long been used to partially ablate resident bone marrow (and other) stem cells and ensure the survival of mice (Figure 3A) [23]. In the classical model we performed fractional irradiation and then allowed a 30-day period before wounding. During the resting period between irradiation and wounding, much of the depletion in the bone marrow compartment would be expected to have recovered with engraftment of the administered bone marrow cells (as demonstrated by FACS analysis). The wounds in this model, however, were delayed in closure compared to control mice indicating that there still was a localized cutaneous radiation effect. In the reverse model, wounding was performed between the first and second doses of radiation. Wound closure in these animals was significantly slower than in the classical model. This was not entirely unexpected, as a recovery period was not allowed prior to wounding.

It is also probable that further irradiation following the stimulatory effects of acute wounding led to additional depletion of local stem and progenitor cells that might not have been so susceptible to radiation in resting skin. The more acute effects of depletion in the bone marrow compartment in the reverse model also cannot be ignored. It is, however, remarkable that wound closure was still delayed more than two weeks after irradiation when recovery in the bone marrow compartment should be expected to be occurring. In many ways it was felt that the reverse model shared phenotypic changes seen in chronic wounds, including depletion of resident stem cells, abnormalities in bone marrow function and delayed healing. We, therefore, chose to test the ability of WBM, BMC and BM-MSC to improve wound closure in the reverse model.

WBM, BMC or BM-MSC was given to reverse model treated mice by tail vein injection 24 hours after the second radiation dose. There was an improvement in wound closure for all cell preparations over the control (untreated mice). As in previous experiments, however, WBM appeared to be superior in achieving a more rapid wound closure than were cultured cells. Also similar to previous experiments, BMC appeared to have an advantage over BM-MSC. A criticism for the reverse model could be that WBM and BMC contained cells more capable of restoring the bone marrow compartment than BM-MSC. This, however, does not appear to fully explain the benefit of administering a mixed bone marrow cell population such as WBM or BMC as these preparations performed better in both angiogenesis and migration assays. It is also very unlikely that significant reconstitution of the bone marrow compartment could have taken place in the first seven days when WBM and BMC showed their greatest effect. What the reverse model experiment may be indicating is the importance of mixed bone marrow stem and progenitor cells in a setting where they are locally and systemically impaired, such as in the case of patients with multiple comorbidities and poorly healing wounds.

Bone marrow derived cells are certain to play many roles in the wound healing process. One role is based on the capacity of bone marrow cells to secrete a variety of growth factors and cytokines that are required to orchestrate tissue repair and regeneration. This mechanism would regulate cellular processes, such as chemotaxis, cell proliferation, angiogenesis, extracellular matrix production and remodeling. Another role is for bone marrow derived cells to differentiate into multiple cell types, including fibroblast myofibroblasts and myoblasts after migrating to the injury site [25]. The use of different types of stem cells has emerged as a promising strategy in regenerative medicine.

Multiple candidate cells have been used in preclinical animal models and in humans to repair or regenerate the injured tissue either directly or indirectly (through paracrine effects), including: embryonic stem cells, induced pluripotent stem cells, endothelial stem cells, bone marrow mononuclear stem cells, and mesenchymal stem cells. Some reports suggest that certain bone marrow derived cells, such as mesenchymal stem cells, secrete a broad spectrum of angiogenic or anti-apoptotic cytokines, which play a role in the process of tissue repair and that BM-MSC conditioned medium has been shown to contain the majority of human cytokines fundamental in wound healing [26,27]. The faster wound closure in WBM compared with BMC and MSC observed in our studies could suggest a higher number of cytokines and growth factors in these cells, which are essential in tissue repair. The results of these experiments support the use of a mixture of cells types in designing clinical approaches to wound healing. Nevertheless, future studies to characterize and compare the cytokines and cell types present in these cells should be done.

## Conclusions

We have shown that WBM is more efficient compared to BMC and MSC in stimulating angiogenesis *in vitro*, inducing fibroblast migration by paracrine effects and in reducing wound size in a murine model of delayed wound healing. Our studies demonstrate that mixed bone marrow cell preparations may be superior to a more purified stem cell formulation in stimulating wound healing.

## Abbreviations

BMC: whole bone marrow cultured cells
BMDC: bone marrow derived cells
BM-MSC: bone marrow-mesenchymal stem cells
BMT: bone marrow transplantation
ECM: extracellular matrix
FACS: fluorescence-activated cell sorting
FBS: fetal bovine serum
GFP: green fluorescent protein
HUVEC: human umbilical vein endothelial cells
MSC: mesenchymal stem cells
NC: negative control
SDF1: stromal derived factor-1
WBM: whole bone marrow
